# The Prevalence of Blood Groups in Domestic Cats in the Saskatoon and Calgary Areas of Saskatchewan and Alberta, Canada

**DOI:** 10.3389/fvets.2020.00160

**Published:** 2020-04-21

**Authors:** Fergal M. McDermott, Stephanie Maloney, Chantal McMillan, Elisabeth Snead

**Affiliations:** ^1^Department of Small Animal Clinical Sciences, Western College of Veterinary Medicine, University of Saskatchewan, Saskatoon, SK, Canada; ^2^Department of Veterinary Clinical and Diagnostic Science, Faculty of Veterinary Medicine, University of Calgary, Calgary, AB, Canada

**Keywords:** blood typing, hemolysis, erythrocyte antigen, neonatal isoerythrolysis, feline

## Abstract

The purpose of this study was to evaluate blood types of domestic cats in two cities in Western Canada (Saskatoon, Saskatchewan and Calgary, Alberta), as well as to determine the risk of mismatched transfusion and neonatal isoerythrolysis. Several cat studies around the world have shown variability in the prevalence of blood types in domestic and pedigree cats. Canadian data based on feline blood types is based out of Montreal. In this study the cohort of cats revealed a higher than anticipated prevalence; of 5% type B and 0.6% AB blood types. In our study, blood typing was performed in 400 domestic cats; 200 in Saskatoon and 200 in Calgary. Blood typing was performed using the gel tube method and the risk of transfusion mismatch (MT) was estimated by adding the risk of a major transfusion reaction and the risk of a minor transfusion reaction. The risk of neonatal isoerythrolysis (NI) was estimated according to the equation (p^2^)(q^2^) + 2pq(q^2^), with q being the b allele frequency and *p* = 1 – q. There was an identical frequency for feline blood types in both Saskatoon and Calgary cats, with 96% type A, 4% type B, and 0% AB. Based on these percentages, the risks of MT and NI in domestic cats were 7.6 and 4 % respectively. The frequency of type B cats in the population was similar to that in the previous Canadian study. These results demonstrate regional differences in prevalence of type B blood in domestic shorthairs across the world and serve to reinforce recommendations to blood type prior to transfusion or mating.

## Introduction

The feline AB blood grouping system was first described in 1981 (1). The blood types differ depending on specific red blood cell (RBC) antigens on their erythrocyte membrane—Type A cats only have “A antigen” and Type B cats only have “B antigen” (1). Type A cats have low titres of weak IgG anti-B antibodies, whereas all type B cats over 3 months of age have high titres of strong IgM anti-A antibodies (2). Type AB cats have neither anti-A or anti-B antibodies in circulation (2). It is proposed that the A and B red blood cell antigens are sialic *N*-glycolyl- and *N*-acetyl-neuraminic acids, respectively (2, 3). Genetic mutations identified in the cytidine monophosphate-N-acetylneuramic acid hydrolase (*CMAH*) gene have been associated with types A, B and AB, but the specific functional effects of these variations have not yet been determined (4, 5).

Type A is the most common blood type in felines worldwide, with the prevalence in most countries between 85 and 100% in non-pedigree cats (1). The prevalence of feline blood types has been shown to vary depending on the breed of cat and the geographical location (6–17).

While the AB system is the most important feline blood system, cross matching incompatibilities and transfusion reactions have occurred in spite of correct AB group matching, leading to assumption that other blood groups exist (18–22). In 2007, a novel (Mik) antigen was discovered on the erythrocytes of 4 cats, and alloantibodies to these non-AB antigens have been shown to be both naturally occurring (19, 20) and produced post transfusion (23).

Clinically, it is the aforementioned strong anti-A alloantibodies that are responsible for potentially fatal haemolytic reactions, the severity of which is more closely related to the alloantibody titre in the type B recipient's blood than with the amount of antigen administered (13, 24–27) In contrast; the weak anti-B alloantibodies in type A cats blood produces minor transfusion reactions which shorten survival of transfused RBC's (11, 24–26).

Type A or type AB kittens born to a type B queen are at risk for NI and therefore severe life-threatening reactions due to anti-A antibodies present in the colostrum within the first 24 h (27). The severity of clinical signs will depend on the antibody titres of the queen, the antibody levels in the colostrum or milk, and the amount of colostrum absorbed by the kitten, meaning not all kittens may be affected equally (24, 27).

Risks of a transfusion reaction and neonatal isoerythrolysis in cats depends on the proportion of both anti-A and anti-B alloantibodies in the population and therefore on the prevalence of blood types in one region. Prior to 2014, information regarding the frequency of feline blood types in Canada had been extrapolated from studies conducted in domestic cats in the 1990s in the United States (7, 15). The first investigation into feline blood type prevalence in Canada was performed in Quebec in 2014 (15), and the results revealed the frequency of type B in domestic and pedigree cats (5%) to be twice the expected rate based on studies in the United States in the 1990's (11, 14). In order to understand what the relative risk of a transfusion reaction or neonatal isoerythrolysis occurring is in our geographical areas we conducted a prospective study to determine the prevalence of feline blood types in domestic cats in the Saskatoon and Calgary. Using this data, we calculated the risk of a mismatched transfusion and for neonatal isoerythrolysis occurring in these locations. We hypothesized that the prevalence of feline blood type B in domestic short hair cats in Western Canada will be higher than previously reported in the studies from the US and would be similar to that in the Quebec study, and that the AB blood type would have the lowest prevalence.

## Materials and Methods

Approval was obtained for the study from the Animal Research Ethics Board and the Veterinary Sciences Animal Care Committee (AC18-0172) at the University of Saskatchewan and the University of Calgary, respectively. The sample size was estimated assuming a prevalence of 2% of type B cats in the feline population based on results obtained in previous North American prevalence studies (11, 14). Therefore, we aimed to enroll 200 cats [95% confidence interval (CI); 2% margin of error] at each primary site. The pool of participants was made up of domestic cats presenting to the veterinary teaching hospital at Western College of Veterinary Medicine (WCVM) in Saskatoon, Saskatchewan, and several local veterinary practices in the region of Calgary, Alberta. All clients signed a consent form to have their cat participate in the study. Data was collected for each cat including signalment, and current health status and health history of the animal. Cats were excluded from being in the study if they had a prior history of receiving a blood transfusion.

Whole blood samples of 0.5 to 1 mL was collected into Ethylenediamine tetra-acetic acid (EDTA) tube from the jugular or medial saphenous vein (appropriately diluted) and the blood typing was performed within 24 h of sample collection. If typing could not be performed immediately after sample acquisition, samples were stored at 4°C between the time of collection and typing. Cats were typed using the standardized 3 tube technique using the commercially available Rapid-H (DMS Laboratories, Inc, Flemington, NJ, USA) gel tube desk-top blood typing kits (28). Back-typing through a reference laboratory was part of the protocol for any potential AB cats, however, since no cats of this blood type were identified this was not pursued.

The Rapid-H gel tube blood typing test relies on agglutination to display reactions consistent with Type A, B, or AB blood (28). It has an overall accuracy of 98.5%, which is higher than other commercially available blood typing methods including card and gel techniques (29, 30). For the Rapid H gel test an aliquot of EDTA anticoagulated feline whole blood was pipetted into the diluent tube, and was inverted several times to ensure uniform mixing. An aliquot from this diluent tube was then pipetted into each of the positive control and the reaction tubes, and all tubes were then incubated at room temperature for 5 min. The tubes were then placed in a compatible fixed-angle rotor centrifuge at the appropriate rpm and time for the model used, as per manufacturer instructions (list provided by Rapid-H). Samples were then removed and the blood type determined according to the manufacturer's instructions by the primary investigators (FMcD,SM), and the results recorded.

The risk of a life-threatening, major transfusion reaction (MTR) following an unmatched transfusion can be calculated as the percentage of type B cats multiplied by the percentage of non-type B cats (type A + type AB) in the population (8, 11, 16). With no AB cats were identified in this study the chance of a minor transfusion (mTR) reaction occurring in these two cities (reduced red cell life span due to incompatibility between a type B donor and type A recipient) was calculated as the percentage of type B cats multiplied by the percentage of type A cats. Also, because no AB cats were identified, the MTR and mTR calculations were identical. The overall risk of a mismatched transfusion (MT) owing to incompatibility in the AB system is the combination of the risks of MTR and mTR. The estimated mating risk for NI was calculated also as previously described (8, 11, 16). The b allele frequency (q) was first calculated assuming a Hardy–Weinberg equilibrium, p^2^ + 2pq + q^2^ = 1 with p = 1 – q; q^2^ = proportion of type B cats. The proportion of mating risk is (p^2^)(q^2^) + 2pq(q^2^). It is important to note that these formulae do not include type AB cats and should our study have identified any type AB cats, this calculation would be less accurate.

## Results

### Saskatchewan Results

Healthy domestic shorthair cats (*n* = 200) were enrolled in the study with an average age of 3.25 y (ranged from 0.25 to 14 y). The population sample consisted of 96 (48%) females and 104 (52%) males. Of these cats, there were 92 sterilized females, 4 intact females, 97 castrated males, and 7 intact males. Cats (*n* = 95) were enrolled through staff and students of, or patients presented to the WCVM VTH. In addition, 31 cats were recruited from local shelters, either presented to the WCVM for elective spay/neuter procedures or strays boarding in the hospital whilst awaiting a foster. Finally, 74 cats came from two low cost, intensive spay/neuter drives organized through the university.

The prevalence rates of feline blood types in the population were: 96% type A cats (*n* = 192), 4% type B (*n* = 8), and 0% type AB (*n* = 0). Of the type B cats identified in this study, none were known to be related.

### Alberta Results

As with Saskatoon, 200 healthy domestic cats were enrolled in the study which had an average age of 3.79 y (ranged from 0.25 to 18 y). The sample consisted of 89 (44.5%) females and 111 (55.5%) males. There were 86 sterilized females, 3 intact females, 109 castrated males, and 2 intact males. In Calgary, 54 cats were enrolled through the primary hospital (VCA Western Veterinary Emergency Centre) and the UCVM; 57 cats were recruited from local shelters and finally 89 cats were enrolled through local general and feline - only clinics within Calgary.

The overall prevalence rates of feline blood types in the Calgary population were as follows: 96% type A cats (*n* = 192), 4% type B (*n* = 8), and 0% type AB (*n* = 0). As with Saskatoon, none of the type B cats identified in this study were known to be related or from the same household.

### Overall Results

The overall population of domestic cats (*n* = 400) enrolled in both Saskatoon and Calgary, revealed an average age of 3.47 y (ranged from 0.25 to 18 y) and consisted of 185 (46.25%) females, 215 (53.75%) males. The prevalence rates for feline blood types in domestic species in both Saskatoon and Alberta were 96% type A (*n* = 384) and 4% type B (*n* = 16). As prevalence for both areas was identical, the mismatched transfusion risks for both Saskatoon and Alberta were identical. The risk of MTR at 3.8% and the risk mTR was 3.8% for both cities. Therefore, the risk of Mismatched Transfusion was 7.6% for both cities and the proportion of mating risk for NI was 4%.

## Discussion

To our knowledge, this is the second prevalence study of feline blood types in Canada, and the first in Western Canada. In the Montreal study in 2014 (15), the prevalence of blood types in domestic and pedigree cats was 95.2% type A, 4.4% type B, and 0.48% type AB. Unlike Montreal, the Saskatchewan and Alberta investigations were solely performed on domestic cats, although our results were almost identical with 96% type A cats, 4% type B, and 0% type AB. The results from all three of these Canadian urban areas demonstrate a higher percentage of type B cats than the previous 1.7% reported in North America (11, 14). In these studies, in domestic cats only, there was a range of between 0 and 1.7% of type B cats. Our study found that for domestic cats only in both Saskatchewan and Alberta, the number of type B cats was twice as high as those studies would have predicted. Further studies in other urban areas across Canada would be interesting to investigate whether this prevalence is consistent throughout the country.

It is important to note that these previous studies (11, 14) from which we base our assumptions on North American prevalence rates are nearly 30 years old. Given that this study illustrates that the prevalence of feline blood types is similar in different regions of Canada, it is possible that current American studies could yield similar results as ours if repeated. It cannot be assumed that similar prevalence rates exist throughout the continent of America, as studies in Australia illustrated a 10% difference in type B prevalence in crossbred cats between Brisbane and Sydney; two cities ~900 km apart (1, 8). Importation of pure and domestic cats with more global travel of families, along with crossbreeding in cats over the years may have contributed to the change in distribution of blood types over time. Neonatal isoerythrolysis, can in theory result in natural selection of type B kittens by the type B female and may also have played a role in the rise in type B cats. As such, novel investigations into feline blood types and their current prevalence in the Americas would be interesting to determine changes in prevalences in feline blood types in different areas and in the same areas over time.

Based on results from a recent study (16) in which risk of mismatched transfusion and neonatal isoerythrolysis risks per country were calculated, the risk of MT and NI in Saskatoon and Alberta are relatively low when compared to other countries at 7.6%, and 4% respectively. This compares to a much higher risk of MT and NI in Australia (8) of 45.3% and 23%, respectively. Interestingly, the results from Saskatoon and Alberta lie between the results from Montreal (MT 9.5%; NI 4.8%) and Rio de Janeiro (MT 5.5%; NI 2.8%), which are the only two blood type prevalence studies to have been performed in the Americas in the last 30 years (15, 17).

This study carries some limitations. In our studied population, none of the cats were pedigree, thus this prevalence is not strictly speaking truly representative of the general cat population in these regions. However, our purpose was to evaluate the most common breed of owned cats in our regions and the importance of blood typing. Large numbers of each pedigree cats of each breed would be needed to determine blood type prevalence of specific breeds within our regions. The Mik antigen blood type was not investigated in this study as such blood typing methods are not readily available. Unlike the previous study performed in Canada, no AB cats were found. This could be due to the overall low worldwide prevalence of AB cats. Furthermore, the presence of type AB cats is most likely to be expected in populations with a higher type B prevalence (31). Due to the variation of appearances of type A results in the test kits used and their similarity to the appearance of a type AB result, it is also possible that an AB cat could have been missed.

This study supports the previous findings of a higher than expected prevalence of type B cats in Canada, within a sample of cats from Saskatchewan and Alberta. Given that this is the second such study, it illustrates a need for more current investigations throughout North America. Since NI and transfusion reactions are potentially fatal, the findings of this study reinforce the importance of typing cats for breeding purposes and typing and cross-matching prior to blood transfusions.

## Data Availability Statement

The datasets generated for this study are available on request to the corresponding author.

## Ethics Statement

The animal study was reviewed and approved by Animal Research Ethics Board, University of Saskatchewan. Written informed consent was obtained from the owners for the participation of their animals in this study.

## Author Contributions

FM and SM share equal contribution toward the investigations undertaken and the writing of this manuscript. CM and ES contributed equally to the revisions of the manuscript.

## Conflict of Interest

The authors declare that the research was conducted in the absence of any commercial or financial relationships that could be construed as a potential conflict of interest.
